# Role of HIV co-receptor usage in viral pathogenesis and cure

**DOI:** 10.1186/1742-4690-10-S1-O43

**Published:** 2013-09-19

**Authors:** Monique Nijhus

**Affiliations:** 1 University Medical Center Utrecht, Netherlands

## 

Binding of the viral envelope glycoprotein gp120 to a co-receptor (CCR5 or CXCR4) is essential for HIV entry into CD4+ host cells. HIV co-receptor tropism is mainly determined by the third hypervariable loop of the viral envelope (gp120-V3). The presence of positively charged amino acids in this V3-loop, especially at positions 11 and 25, is associated with binding to a negatively charged region of the CXCR4 co-receptor, whereas a more neutrally charged V3-loop is associated with CCR5 co-receptor binding.

The majority of HIV infections appear to be due to CCR5-using ‘R5’ virus, which continues to predominate during the course of the infection. As time progresses however, about 50% of patients have a shift in their viral population to include a growing amount of CXCR4-using ‘X4’ virus. Although an R5 to X4 switch is strongly correlated with increased rates of disease progression, it remains largely unknown which factors are causing the switch. Viral and immunological factors involved in co-receptor switching will be discussed as well as the role of co-receptor usage in HIV cure strategies.

